# Sex difference in prebiotics on gut and blood–brain barrier dysfunction underlying stress‐induced anxiety and depression

**DOI:** 10.1111/cns.14091

**Published:** 2023-01-17

**Authors:** Jiajun Jiang, Yaoyang Fu, Anying Tang, Xingle Gao, Danhua Zhang, Yuting Shen, Tingting Mou, Shaohua Hu, Jingfang Gao, Jianbo Lai

**Affiliations:** ^1^ Department of Psychiatry, The First Affiliated Hospital Zhejiang University School of Medicine Hangzhou China; ^2^ The First Affiliated Hospital of Zhejiang Chinese Medical University (Zhejiang Provincial Hospital of Traditional Chinese Medicine) Hangzhou China; ^3^ The Key Laboratory of Mental Disorder's Management in Zhejiang Province Hangzhou China; ^4^ Brain Research Institute of Zhejiang University Hangzhou China; ^5^ Zhejiang Engineering Center for Mathematical Mental Health Hangzhou China; ^6^ Department of Neurobiology, NHC and CAMS Key Laboratory of Medical Neurobiology, School of Brain Science and Brian Medicine, and MOE Frontier Science Center for Brain Science and Brain‐machine Integration Zhejiang University School of Medicine Hangzhou China; ^7^ The First College of Clinical Medicine Zhejiang Chinese Medical University Hangzhou China

**Keywords:** blood–brain barrier, gut microbiota, prebiotic, sex difference, stress

## Abstract

**Background:**

Most of the previous studies have demonstrated the potential antidepressive and anxiolytic role of prebiotic supplement in male subjects, yet few have females enrolled. Herein, we explored whether prebiotics administration during chronic stress prevented depression‐like and anxiety‐like behavior in a sex‐specific manner and the mechanism of behavioral differences caused by sex.

**Methods:**

Female and male C57 BL/J mice on normal diet were supplemented with or without a combination of fructo‐oligosaccharides (FOS) and galacto‐oligosaccharides (GOS) during 3‐ and 4‐week chronic restraint stress (CRS) treatment, respectively. C57 BL/J mice on normal diet without CRS were used as controls. Behavior consequences, gut microbiota, dysfunction of gut and brain–blood barriers, and inflammatory profiles were measured.

**Results:**

In the 3rd week, FOS + GOS administration attenuated stress‐induced anxiety‐like behavior in female, but not in male mice, and the anxiolytic effects in males were observed until the 4th week. However, protective effects of prebiotics on CRS‐induced depression were not observed. Changes in the gene expression of tight junction proteins in the distal colon and hippocampus, and decreased number of colon goblet cells following CRS were restored by prebiotics only in females. In both female and male mice, prebiotics alleviated stress‐induced BBB dysfunction and elevation in pro‐inflammatory cytokines levels, and modulated gut microbiota caused by stress. Furthermore, correlation analysis revealed that anxiety‐like behaviors were significantly correlated with levels of pro‐inflammatory cytokines and gene expression of tight junction proteins in the hippocampus of female mice, and the abundance of specific gut microbes was also correlated with anxiety‐like behaviors, pro‐inflammatory cytokines, and gene expression of tight junction proteins in the hippocampus of female mice.

**Conclusion:**

Female mice were more vulnerable to stress and prebiotics than males. The gut microbiota, gut and blood–brain barrier, and inflammatory response may mediate the protective effects of prebiotics on anxiety‐like behaviors in female mice.

## Graphical abstract

The gut microbiota, blood–brain barriers, and inflammatory response may mediate the protective effects of prebiotics on anxiety‐like behaviors in female mice.

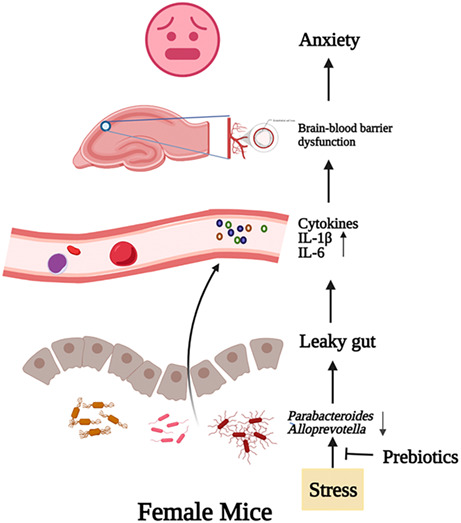

## 1 INTRODUCTION

Depression is one of the major health‐related causes of disability, with heavy burden on patients, families, and society.^1^, ^2^ Pathophysiological underpinnings, such as monoamine hypothesis,^3^ hypothalamic–pituitary–adrenal axis changes,^4^ inflammation,^5^ and other factors, may partially explain the etiology of depression, but the exact mechanisms underlying depression remain elusive.

Recently, emerging studies have revealed that the gut–brain axis may play an important role in depression.^6^ Compared with healthy individuals, the diversity and abundance of microbiota in patients with major depressive disorder (MDD) changed significantly.^7^ Depression‐like and anxiety‐like behavior, presented by forced swimming test (FST), open field test (OFT), and sucrose preference test (SPT), could be transferred by fecal microbiota transplantation (FMT) in rodent studies.^8^, ^9^ In addition, probiotics and prebiotics that could change the composition of microbiota relieved the symptom of depression.^10^ Fructo‐oligosaccharides (FOS) and galacto‐oligosaccharides (GOS) administration reversed anxiety‐like and depression‐like behavior caused by chronic stress in mice.^11^ These studies provided preliminary evidence that gut microbiota might act as the key components of regulating anxiety and depression and become a potential target of treatment in mood disorders.

Immune system is recognized as one predominant pathway connecting the gut microbiota and the brain.^12^ Various metabolites of bacteria inhabiting in the intestinal tract activate the immune cells in the intestinal mucosa, or enter into the peripheral blood to mediate immune response.^13^ Generally, the intestinal barrier helps our body shield pathogens, toxins, and intraluminal antigens from the intestinal flora,^14^ and the blood–brain barrier (BBB) protects the central nervous system from specific components in the peripheral circulation.^15^ However, stress could disrupt the intestinal barrier function by affecting transepithelial electrical resistance, gene expression of tight junction molecules, mast cell density, and mucin O‐glycosylation,^16^, ^17^ permitting pathogens to enter into the circulation, and stimulate immunocytes to produce pro‐inflammatory cytokines.^18^ Modulation of the gut microbiota composition by prebiotics and probiotics administration could increase tight junction protein expression to strengthen the intestinal barrier function.^19^ For example, *Lactobacillus rhamnosus GG* promoted the maturation of intestinal barrier by increasing claudin‐3 expression in neonatal mice,^20^ and GOS increased intestinal tight junction proteins(zo‐1, occluding, and claudin‐1) expression in lipopolysaccharide (LPS)‐challenge mice.^21^ Studies of increasing BBB permeability in germ‐free (GF) mice^22^ and antibiotics‐treated mice^23^ showed that intestinal floras were also involved in regulating the BBB function. Lipopolysaccharide, the main component of gram‐negative bacteria, could activate gastrointestinal immune cells to release pro‐inflammatory cytokines,^24^ which further disrupted the BBB function by downregulating the expression of tight junction proteins.^25^ These findings supported that gut microbiota could participate in the mechanisms of stress‐induced intestinal barrier and BBB dysfunction.

Across the lifespan, females were relatively more vulnerable to depression and anxiety than males, and the prevalence rate was twice that of males.^26^ Circulating gonadal hormones^27^ and genetic sex^28^ might underlie the sex differences in depression and anxiety.^29^ Recently, a clinical study has found that there were different dominant floras between female and male patients with depression.^30^ Animal studies also reported that colonic abundance of microbiota changed in a sex‐specific manner in prenatally stressed offspring at weaning.^31^, ^32^ Probiotics (*L. lactis*, *L. cremoris*, *L. diacetylactis*, and *L. acidophilus*) improved depression‐like and anxiety‐like behavior induced by LPS earlier in female than that in male mice, and LPS‐induced gut microbiota change was reduced by probiotics in a sex‐specific manner as well.^33^ Therefore, gut microbiota might also play a vital role in the sex difference in depression and anxiety.

As nutrients for microorganisms, FOS and GOS could promote the growth of beneficial bacteria (*Bifidobacteria and Lactobacilli*), improve the intestinal barrier function, and reduce stress‐related behavior in males, such as depression‐like and anxiety‐like behaviors.^11^, ^34^ However, few studies involved the prevention and treatment effects of prebiotics on depression and anxiety of females. Chronic stress‐induced gut microbiota differences in sex also needed to be extensively explored. And whether inflammation response, intestinal barrier, and BBB dysfunction induced by chronic stress could be prevented by prebiotics in a sex‐specific manner required extensive elaboration. Therefore, this study aimed to assess sex difference in prebiotics on stress‐induced depression‐like and anxiety‐like behavior, inflammation, and gut and brain barrier dysfunction. First, we evaluated whether prebiotics prevented stress‐induced depression‐like and anxiety‐like behaviors in a sex‐specific manner; then, sex difference in prebiotics on gut microbiota, inflammatory cytokines, and tight junction molecules were measured; finally, we assessed the correlation between stress‐induced behaviors, gut microbiota, and blood–brain barrier.

## 2 METHODS

### 2.1 Animals

**FIGURE 1:**
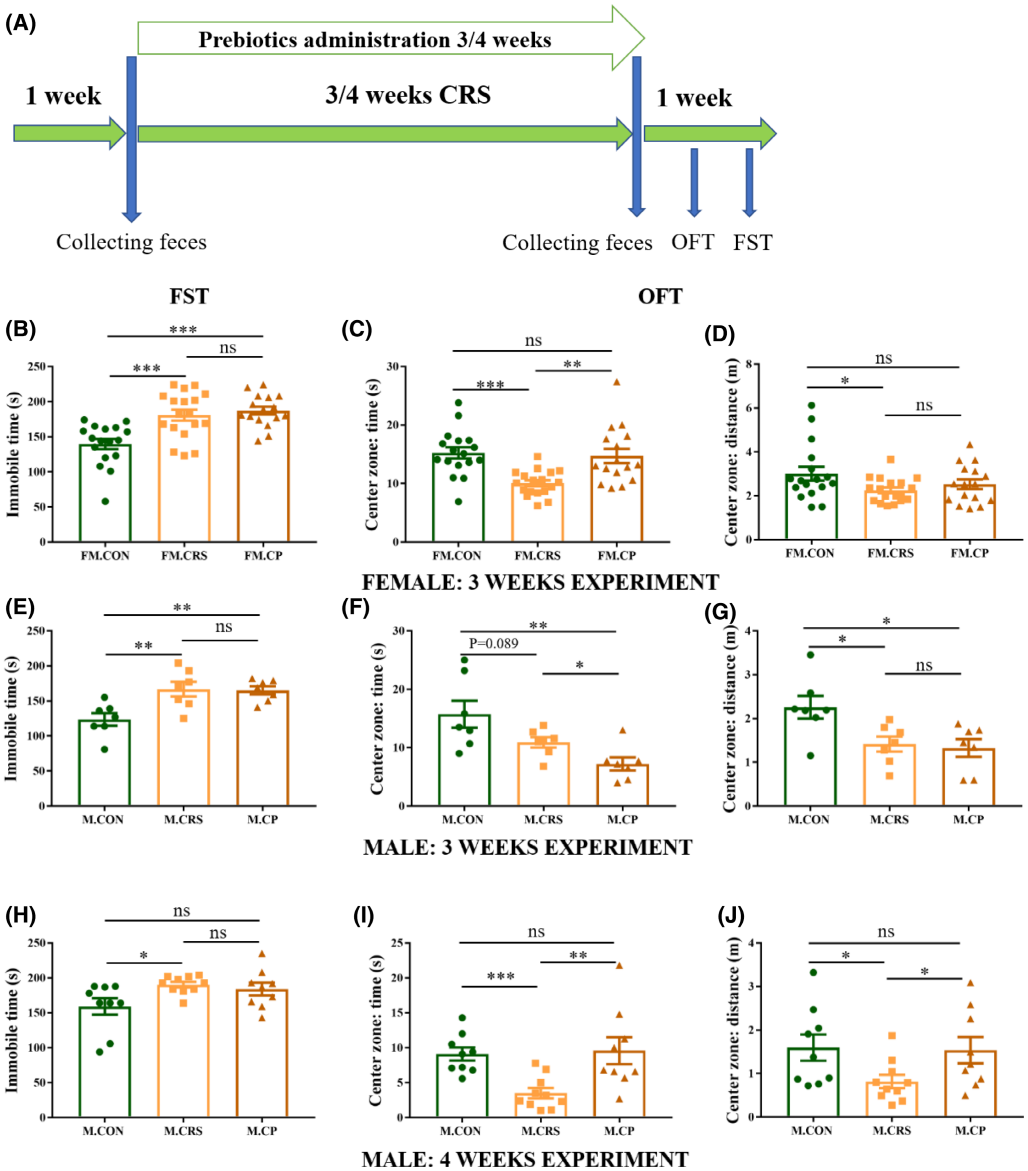
Anxiety‐like and depression‐like behaviors in 3‐ and 4‐week experiment. (A) Mice experimental schedule. (B, E, H) FOS + GOS administration did not affect immobile time in FST, which was increased after 3/4‐week CRS treatment in all mice groups. (C, D) In female mice, FOS + GOS administration prevented decreased the time (but not distance) in center zone of the OFT caused by 3‐week CRS. (F, G) Compared with the CON group, 3‐week CRS in male mice decreased the distance in center zone of the OFT, with a tendency in time. Prebiotics administration further increased the time spent in center zone, with no effect on the distance. (I, J) In 4‐week experiment, male mice in the CRS group showed shorter time and distance spent in center zone of the OFT than that in the CON and CP groups. *p < 0.05, **p < 0.01, ***p < 0.001; Independent Sample *t*‐test analysis (data on depression‐like behavior in female, and immobile time and distance in center zone in 3‐week experiment of male); nonparametric test (data on anxiety‐like behavior in female, time in center zone in 3‐week experiment of male, and depression‐like and anxiety‐like behaviors in 4‐week experiment of male); data represent mean ± SEM.

Female and male C57BL/J SPF mice (aged 6–8 weeks) were housed separately in each polycarbonate cage with ad libitum access to food and water in a 12:12‐h dark–light cycle with temperature at 20 ± 1°C. Female and male mice were fed in separate cages with 5–6 mice in each cage. After a 1‐week period of adaptation, male and female mice were randomly divided into chronic restraint stress (CRS), CRS + prebiotics (CP), and controls (CON) groups, separately. Mice in the CRS group were fed by water and restrained for 4 h every day for 3 consecutive weeks. Mice in the CP group were fed by prebiotics (a combination of FOS and GOS, dissolved in drinking water for 0.3–0.4 g/mouse/day) and restrained for 4 h every day for 3 consecutive weeks. Mice in the CON group were fed by water without stress. All animal experiments (Figure 1A) were approved by the Animal Experimentation Ethics Committee of the First Affiliated Hospital, Zhejiang University School of Medicine.

### 2.2 Behavioral test

Open field test (OPT). All mice were kept in a room with dim light for 2 h to adapt to the environment, and then, each mouse was placed in the center of open field (50 cm × 50 cm × 50 cm) for 5 min. The movement of each mouse was tracked by a video camera and was recorded by the ANY‐maze system. Total distance and distance and time in the center field (25 cm × 25 cm) in each mouse were counted.

Forced swim test (FST). Each mouse was put in a transparent plastic cylinder (12 cm diameter, 25 cm height) of water (23–25°C) gently and was recorded by a video camera for 6 min. Mice heads were allowed to submerged under the water when putting the mice into water. The immobile time for the last 4 min would be counted by a researcher who was blind to the grouping. The definition for immobility is the absence of all movement except for small motions required to remain afloat.

### 2.3 Real‐time PCR

All mice were sacrificed after anesthesia, and proximal colon, distal colon, and hippocampus tissues were extracted on ice and kept at −80°C before testing. Total RNAs from mice tissues were isolated using TRIzol reagent according to the manufacturer's instructions (Invitrogen). Reverse transcription was performed using an ABScript II cDNA First Strand Synthesis Kit (ABclonal) in a 20 ml reaction volume. Genes of interest were detected by QuantStudio 5DXReal‐Time PCR System using SYBR Green Fast qPCR Mix (ABclonal). Each sample was tested three times, and actin was used as an internal control. Primers for β‐actin, zonula occludens 1 (zo‐1), occluding (ocln), claudin‐1 (cldn1), claudin‐2, claudin‐5, claudin‐8, and mucin‐2 (muc‐2) are given in Table S1.

### 2.4 Enzyme‐linked immunosorbent assay (ELISA) analysis

Proximal colon, distal colon, and hippocampus tissues from mice were homogenized in RIPA buffer containing protease inhibitors and then kept on ice for 30 min. The liquid containing protein was centrifuged at 12,000 *g*/min (4°C) for 15 min and supernatant was exacted. BCA protein assays (Beyotime) were used to measure protein concentration. The concentrations of interleukin 1 beta (IL‐1β) and interleukin 6 (IL‐6) were measured by ELISA kits (Cloud‐Clone).

### 2.5 Hematoxylin–eosin (HE) Staining

Colon tissues from female and male mice were exacted on ice and then fixed in 4% paraformaldehyde for 24 h. The fixed colon tissues were dehydrated with different concentration of alcohol, and then, xylene was used to replace the alcohol in tissues. Tissues were embedded in paraffin and cut into 5‐μm sections transversely by a microtome. Paraffin sections were dewaxed with xylene, hydrated with alcohol, and stained with hematoxylin and eosin. A microscope (Eclipse ci‐l; Nikon) was used to select the target area of tissues for 200× imaging. The circumference and the number of goblet cells of five colonic glands in each section were measured by Image‐Pro Plus 6.0 analysis software. The number of goblet cells in colonic gland of unit length could be calculated in each section.

### 2.6 Blood–brain barrier (BBB) permeability test

After 3‐week experiment, mice were injected with 4 ml/kg of 2%(w/v, g/ml) Evans Blue (EB) (CAT No. E2129, Sigma‐Aldrich) via tail veins.^35^ After the dye circulated in the body for 2 h, hippocampus was extracted after 1× PBS cardiac perfusion and weighed, and then incubated in 300 μl formamide at 55°C for 24 h to extract EB from tissues. Centrifuge the mixture at 12,000 g/min for 5 min, and measure absorbance at 610 nm by enzyme marker (BioTek Synergy Neo2). EB concentration was calculated by comparison with the Evans Blue standard curve.

### 2.7 16 S rRNA Sequencing

DNA extraction. Total genomic DNA was extracted from mice feces using DNA Extraction Kit (QIAamp 96 PowerFecal QIAcube HT kit, QIAGEN) following the manufacturer's instructions. Quantification of DNA was tested by the Nanodrop 2000 (Thermo Fisher) and stored at −20°C.

Library construction and sequencing. V3–V4 region of 16 S rRNA was selected for PCR amplification with primers 343 F and 798 R. Amplicon quality was visualized using gel electrophoresis, purified with AMPure XP beads (Agencourt), and amplified for another round of PCR. After purification with the AMPure XP beads, the final amplicon was quantified using Qubit dsDNA Assay Kit (Life Technologies). Equal amounts of purified amplicon were pooled for subsequent sequencing. Sequencing was performed on the Illumina MiSeq according to protocol.

Bioinformatic analysis. Raw sequencing data were in FASTQ format. Paired‐end reads were then preprocessed using Trimmomatic software. Clean reads were subjected to primer sequences removal and clustering to generate operational taxonomic units (OTUs) using Vsearch software with 97% similarity cutoff. The representative read of each OTU was selected using QIIME package.

### 2.8 Statistical analysis

All data were analyzed by SPSS version 26. The results were presented as mean ± standard error of the mean (X ± SEM). Normality of data on behaviors, gene expression of tight function proteins and muc2, inflammatory cytokines, and goblet cells were tested by Shapiro–Wilk test. Normally distributed data were analyzed by independent sample test, and data that did not exhibit a normal distribution were analyzed by nonparametric test. Kruskal–Wallis test was performed to compare alpha diversity and relative abundance in gut microbiota between groups. Pearson and Spearman correlation analyses were used to compare the correlation between gut microbiota, behavioral variables, inflammation profiles, and gene expression of tight function protein levels. The significant difference was set at *p* < 0.05 in this study.

## 3 RESULTS

### 3.1 Prebiotics mitigated anxiety‐like behavior caused by CRS in female in the 3rd week and male mice in the 4th week, but did not alleviate depression‐like behavior

After 3‐week experiment, we tested depression‐like behavior in FST and anxiety‐like behavior in OFT. Both female and male mice in the CRS group showed behavioral despair (immobile time in FST) compared with the CON group, but prebiotics did not show antidepressive effects in both gender mice (Figure 1B,E). In female mice, 3‐week CRS significantly decreased time and distances in the center zone of OFT compared with the CON group, and prebiotics administration during CRS period restored the time in center zone caused by CRS, but had no effect on the distances (Figure 1C,D). In male mice group, mice in both CRS and CP groups moved shorter distances in center zone than those in the CON group, and 3‐week CRS tended to decrease time staying in the center zone compared with the CON group and prebiotics administration during experiment further reduced the time in the center zone significantly in male mice (Figure 1F,G). There was no difference in total distance of OFT among three groups both in female and male mice (Figure S1A–D).

Next, we accessed antidepressive and anxiolytic effects of prebiotics in male mice after 4‐week experiment. Prebiotics also did not rescue behavior despair induced by 4 weeks CRS (Figure 1H). Compared with the CON group, male mice in the CRS group showed less time and distances in the center zone of OFT, which were alleviated by prebiotics (Figure 1I,J). Prebiotics administration during CRS also increased the total distance and decreased the immobile time in OFT significantly, compared with CON and CRS groups (Figure S1E,F).

### 3.2 Prebiotics restored the downregulated muc‐2 expression and the number of goblet cells in colon caused by CRS in female mice, but not in male mice

**FIGURE 2:**
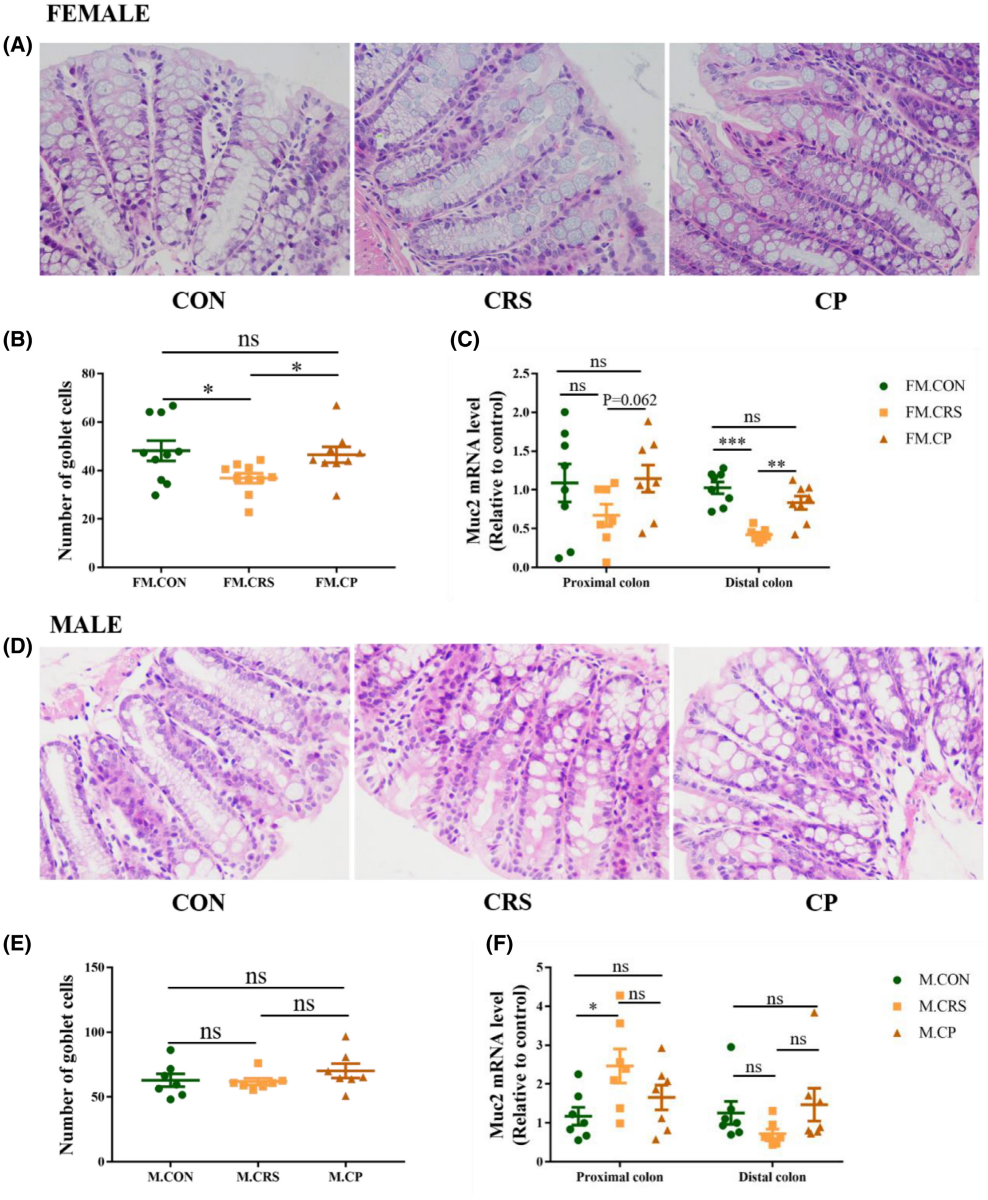
Effects of CRS and prebiotics on number of goblet cells and Muc2 gene expression. (A, D) Representative HE staining images of colon issue in female and male mice. (B, E) GOS + FOS administration prevented the decrease in the number of goblet cells per colonic crypt length caused by 3‐week CRS in female (*n* = 8–10), but there were no differences between male mice groups (*n* = 7). (C) 3‐week CRS treatment decreased Muc2 expression in distal colon of female mice (*n* = 8), which was reversed to the normal level by prebiotics administration. (F) No differences were observed in distal colon between three male mice groups (*n* = 7). All the images were taken under the 400× microscope. FM, female mice group; M, male mice group; *p < 0.05, **p < 0.01, ***p < 0.001; Independent Sample t‐test analysis; nonparametric test (data on muc‐2 gene expression in the distal colon of male); data represent mean ± SEM.

In the proximal colon of female mice, muc‐2 gene expression remained unchanged in the CRS group compared with CON and CP groups. But in the distal colon of female mice, decreased gene expression of muc‐2 was observed in the CRS group compared with the CON group, which could be rescued by prebiotics administration (Figure 2C). In the proximal colon of male mice, CRS increased muc‐2 expression, but prebiotics did not restore the increased muc‐2 mRNA level caused by CRS. No difference in the expression of muc‐2 was observed among the three male groups in the distal colon (Figure 2F).

By analyzing the number of goblet cells in the colon of female mice, we found that the number of goblet cells was decreased in the CRS group compared with the CON group, which could be restored by prebiotics (Figure 2A,B). No statistical difference in the number of goblet cells in the colon was found among male groups (Figure 2D,E).

### 3.3 Three‐week CRS and prebiotics affected colon and brain–blood barrier function in a sex‐specific manner

#### 3.3.1 Gene expressions of tight junction proteins

##### Female mice

**FIGURE 3:**
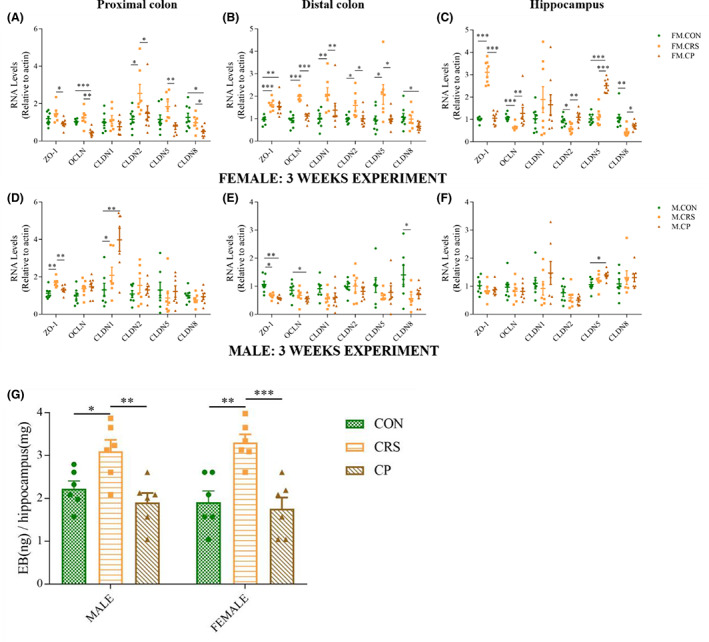
Effects of CRS and prebiotics on gene expression of tight function proteins. (A–C) The gene expression of tight function proteins in the proximal, distal colon and hippocampus of female mice. (D–F) The gene expression of tight function protein in the proximal, distal colon, and hippocampus of male mice. (G) Quantitation of Evans Blue extravasation in hippocampus. Hippocampus was incubated with 300 μl formamide to extract extravasated Evans Blue. Optical density was measured at 610 nm, and the measurements converted into ng dye extravasated per mg tissue. *p < 0.05, **p < 0.01, ***p < 0.001; Independent Sample *t*‐test analysis; nonparametric test (data on gene expression of cldn2 in the proximal colon, cldn2 in the distal colon, and ocln and cldn 1 in the hippocampus of female; data on gene expression of cldn1; and 5 in the proximal, cldn5 in the distal colon, and cldn1 in the hippocampus of male); data represent mean ± SEM.

In the proximal colon, the gene expression of cldn2 increased significantly in the CRS group compared with the CON group, but zo‐1, ocln, cldn2, and cldn8 mRNA levels in the CP group were lower than those in the CRS group (Figure 3A). In the distal colon, increased gene expression of zo‐1, ocln, cldn1, cldn2, and cldn5 was observed in the CRS group compared with the CON group, and prebiotics administration during CRS decreased the gene expression of ocln, cldn1, cldn2, cldn5, and cldn8 compared with the CRS group (Figure 3B). In the hippocampus, increased gene expression of zo‐1 and decreased expression of ocln, cldn2, and cldn8 was showed in the CRS group compared with the CON group, and prebiotics administration during CRS period restored changes in gene expressions caused by CRS (Figure 3C).

##### Male mice

In the proximal colon, increased gene expression of zo‐1 and cldn1 was observed in the CRS group compared with the CON group, and zo‐1 mRNA level in the CP group was similar to that of the CON group (Figure 3D). In the distal colon, gene expression of zo‐1 and cldn8 in the CRS group was lower than that in the CON group, but no statistical difference was detected in the CP group compared with the CRS group (Figure 3E). By analyzing the gene expressions in the hippocampus, there was no significant difference between the CON and CRS groups, or between the CRS and CP groups (Figure 3F).

#### 3.3.2 BBB permeability

After 3‐week experiment, mice were injected with 2% (g/ml) EB solution via the tail vein to assess BBB permeability. Three‐week CRS disrupted BBB permeability in hippocampus, and Prebiotics could alleviate the changes in both female and male mice (Figure 3G).

### 3.4 Prebiotics alleviated CRS‐induced inflammation status in both female and male mice

After 3‐week experiment and behavioral tests, mice were sacrificed with colon and brain tissues extracted on ice. To assess whether prebiotics could alleviate inflammation levels caused by CRS, we measured IL‐1β and IL‐6 levels in the proximal colon, distal colon, and hippocampus by ELISA.

**FIGURE 4:**
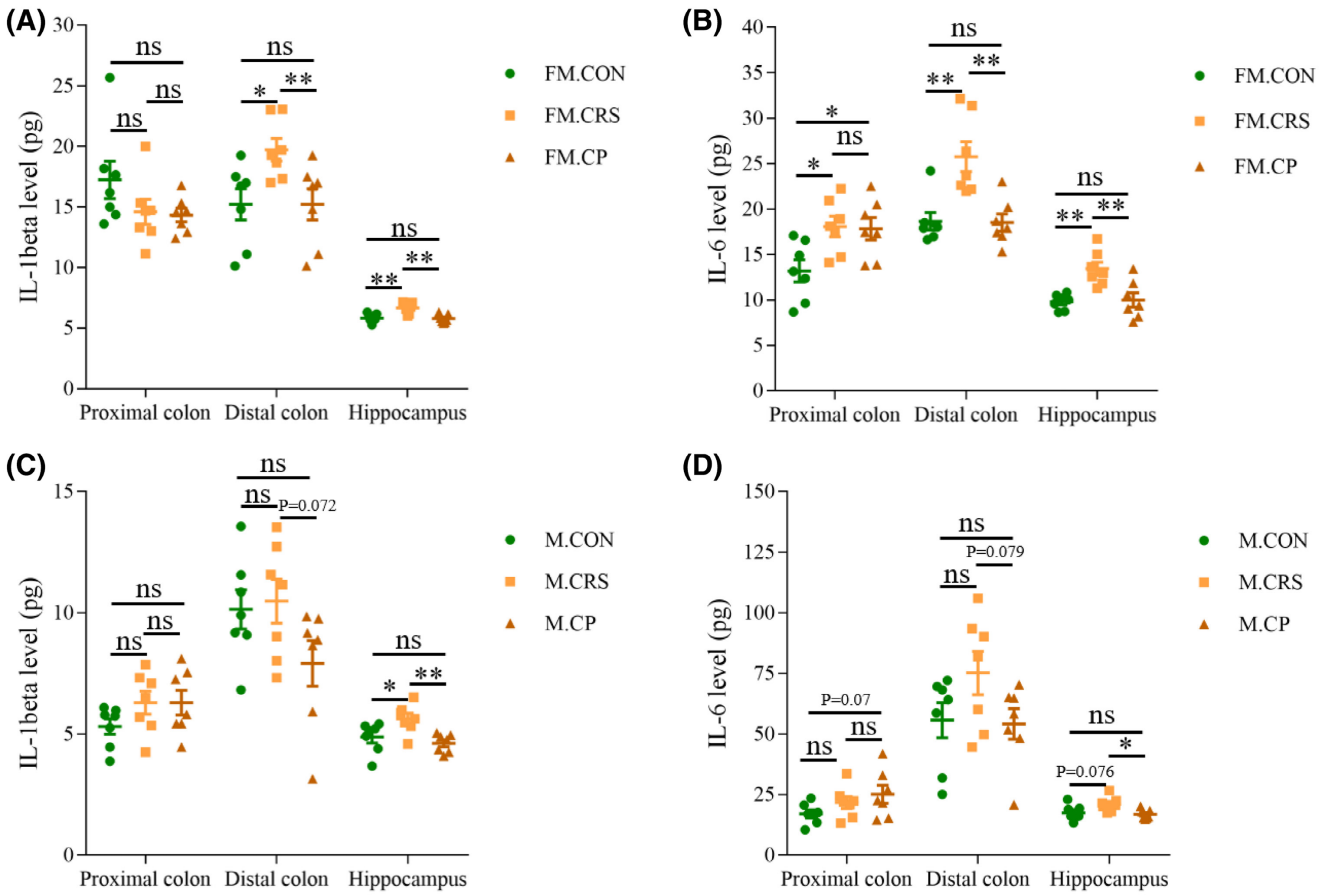
Effects of CRS and prebiotics on pro‐inflammatory cytokines in the colon and hippocampus. (A, B) In female mice, prebiotics prevented the increase in IL‐1β and IL‐6 levels in the distal colon and hippocampus caused by 3‐week CRS, but not in proximal colon. (C, D) 3‐week CRS treatment increased IL‐1β levels and had a tendency to increase IL‐6 levels in the hippocampus of male mice, which were alleviated by prebiotics administration during 3‐week experiment. *n* = 7–8; *p < 0.05, **p < 0.01, ***p < 0.001; Independent Sample t‐test analysis; nonparametric test (data on IL‐1β level in the proximal colon and IL‐6 level in the distal colon of female); data represent mean ± SEM.

In the female mice group, IL‐6 levels in the proximal colon, distal colon, and hippocampus significantly were increased in the CRS group compared with the CON group, and prebiotics administration during 3‐week CRS downregulated IL‐6 levels in the distal colon and hippocampus, but not in the proximal colon (Figure 4B); IL‐1β levels in the distal colon and hippocampus were also increased significantly after 3‐week CRS, which were downregulated to the level of the CON group following prebiotics administration during 3‐week CRS treatment, but no significant difference was found in the proximal colon among three groups (Figure 4A).

In the male mice group, 3‐week CRS increased IL‐1β levels, with an upward trend of IL‐6 levels in the hippocampus, but not in the proximal and distal colon (Figure 4C,D). Compared with the CRS group, prebiotics administration reduced IL‐1β and IL‐6 levels in the hippocampus (Figure 4C,D).

### 3.5 Three‐week CRS and prebiotics administration modulated the relative abundance of gut microbiota in a sex‐specific manner

**FIGURE 5:**
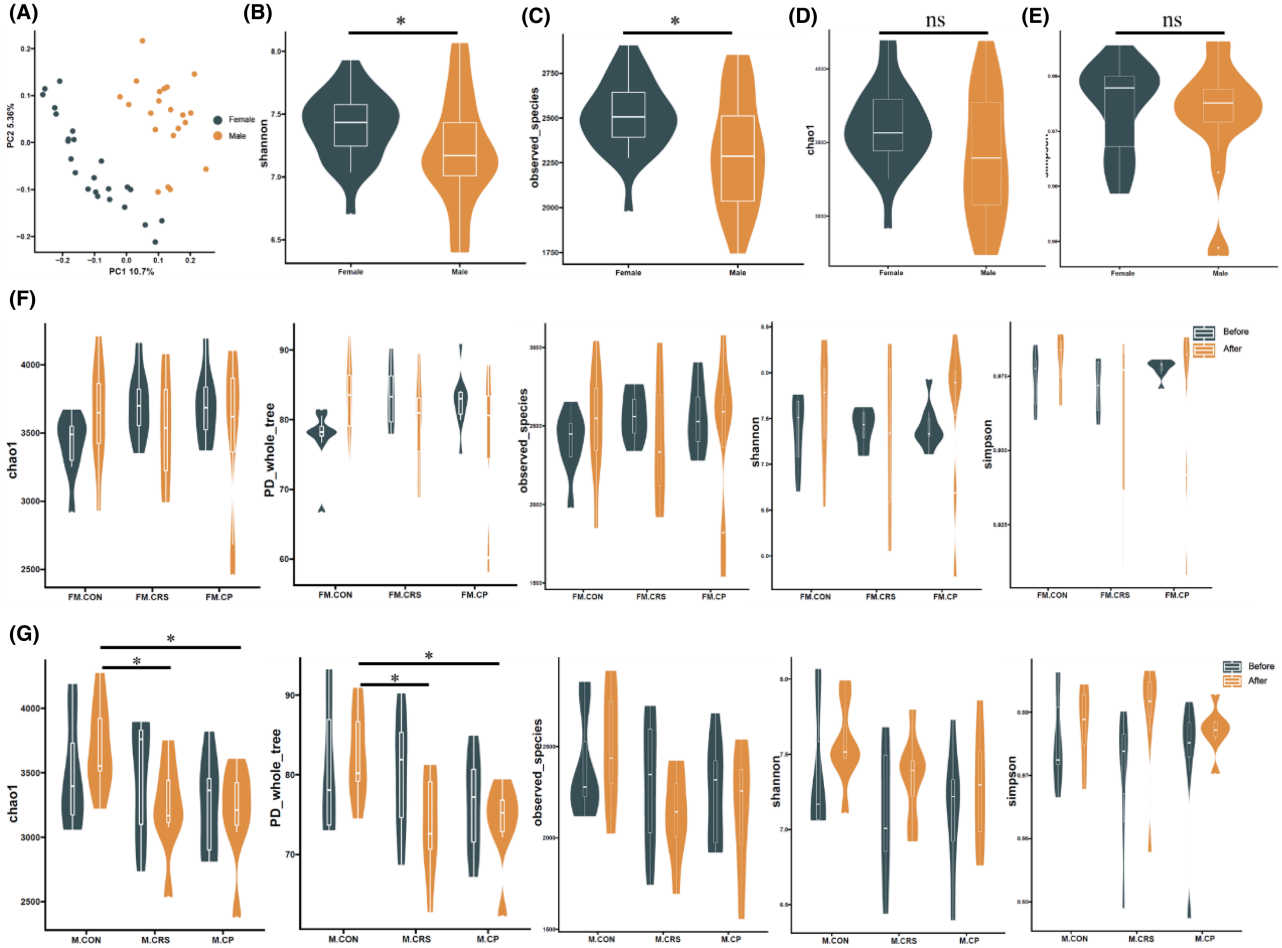
Results of α diversity and PCA analysis in gut microbiota between female and male mice groups. (A) The result of PCA showed the difference in microbiota distribution between female (*n* = 24) and male (*n* = 21) mice before experiment. (B–E) Shannon and observed species indices in female mice (*n* = 24) were higher than that in male mice (*n* = 21) before experiment. (F) There were no differences in alpha diversity between three female mice groups after 3‐week experiment (*n* = 8). (G) In male mice, Chao1 and PD whole tree indices were higher in the CON group (*n* = 7) than these in the CRS (*n* = 7) and CP (*n* = 7) groups after experiment. *p < 0.05; Kruskal–Wallis test and Mann–Whitney test.

At baseline, principal coordinate analysis (PCoA) showed a clear separation in microbial population between female and male mice (Figure 5A). In regard to the alpha diversity of microbiota, Shannon and ‘observed species’ indices in female were higher than that in male mice (Figure 5B,C).

In female mice, there were no differences in the alpha diversity between three groups after experiment (Figure 5F). In male mice, the indices of Chao1 and ‘PD whole tree’ in the CON group were significantly higher than those in the CRS and CP groups after 3‐week experiment (Figure 5G).

**FIGURE 6:**
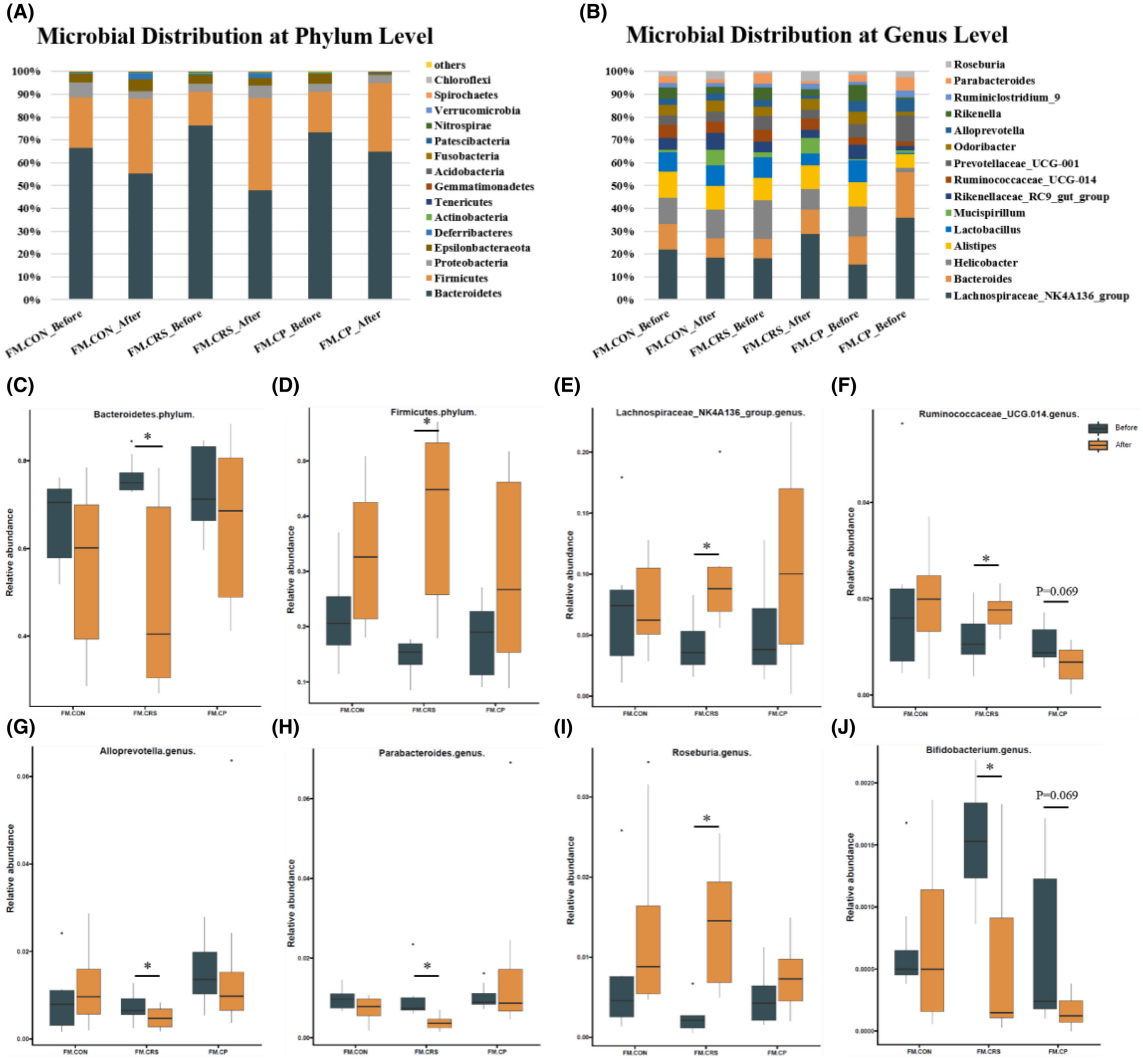
Microbial distribution and relative abundance of selected phylum and genus among female mice groups. (A, B) Microbial distribution at phylum and genus levels among female groups. *Bacteroidetes* and *Firmicutes* were the main components of microbiota at phylum level, and *Lachnospiraceae_NK4A136_group* and *Bacteroides* were the dominant microbial group at asgenus level. (C, D) At phylum level, relative abundance of *Bacteroidetes* and *Firmicutes* changed significantly in the CRS group after 3‐week experiment, but not in the CON or CP groups. (E–J) At genus level, relative abundance of *Lachnospiraceae_NK4A136_group*, *Ruminococcaceae_UCG‐014*, and *Roseburia* increased, and the abundance of *Alloprevotella* (*p* = 0.05), *Bifidobacterium* (*p* = 0.036), and *Parabacteroides* (*p* = 0.012) decreased significantly after 3‐week CRS treatment, but not in the CON or CP groups. *p < 0.05; Kruskal–Wallis test and Mann–Whitney test; *n* = 7–8.

By analysis of relative abundances at phylum levels in female mice, *Bacteroidetes* and *Firmicutes* were the main components of microbiota (Figure 6A), showing significant change in the CRS group after experiment (*Bacteroidetes*, *p* = 0.025; *Firmicutes*, *p* = 0.012), but there was no significant change in the CON or CP groups (Figure 6C,D). In male mice group, *Bacteroidetes* and *Firmicutes* were also the dominant microbiota (Figure S2A), showing no significant change in all groups after 3‐week experiment (Figure S2C,D); but 3‐week CRS treatment decreased the abundance of *Epsilonbacteraeota* (*p* = 0.018), which was not observed in the CON or CP groups (Figure S2E).

In female and male mice, *Lachnospiraceae_NK4A136_group* and *Bacteroides* were the dominant microbial group at the genus level (Figure 6B; Figure S2B). In female mice, the abundance of *Lachnospiraceae_NK4A136_group* (*p* = 0.012), *Ruminococcaceae_UCG‐014* (*p* = 0.05), and *Roseburia* (*p* = 0.012) increased, and the abundance of *Alloprevotella* (*p* = 0.05), *Bifidobacterium* (*p* = 0.036), and *Parabacteroides* (*p* = 0.012) decreased significantly after 3‐week CRS (Figure 6E–J). Among these genera mentioned above, no bacteria changed significantly in the CON or CP groups. In male mice, decreasing abundance of *Helicobacter* (*p* = 0.018), *Rikenellaceae_RC9_gut_group* (*p* = 0.028), and *Bifidobacterium* (*p* = 0.018), and increasing abundance of *Roseburia* (*p* = 0.043) was observed in the CRS group after 3‐week experiment, and similar changes occurred in the abundance of *Rikenellaceae_RC9_gut_group* (*p* = 0.018) and *Roseburia* (*p* = 0.018) in the CP group (Figure S2H–K). We also found that the abundance of *Lachnospiraceae_NK4A136_group* (*p* = 0.018) and *Bacteroides* (*p* = 0.018) changed in the CON group after 3‐week experiment, but not in the CRS and CP groups (Figure S2F,G).

### 3.6 The abundance of gut microbiota was correlated with behaviors, inflammation condition, and gut and brain barrier function in female mice

**FIGURE 7:**
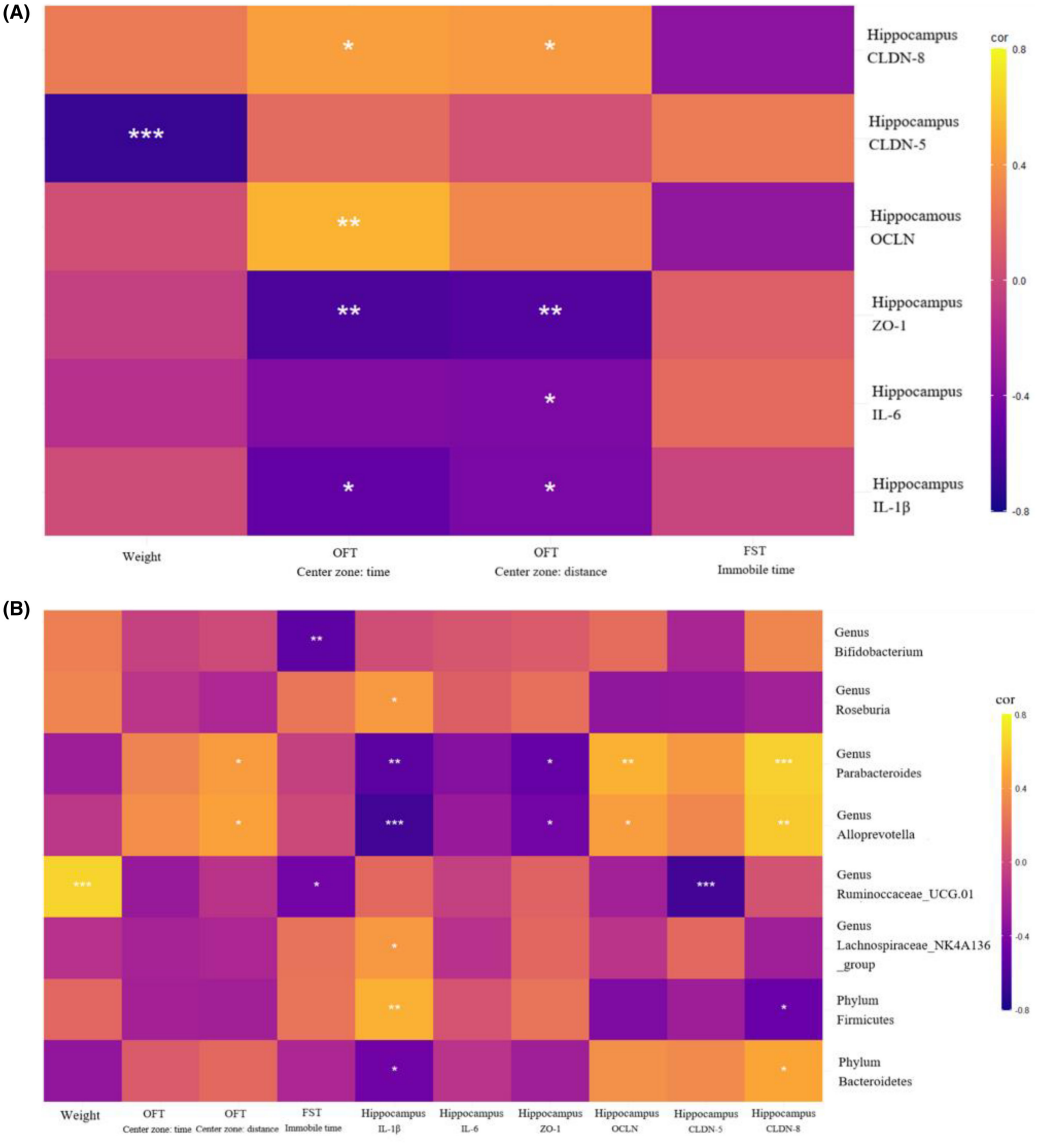
Correlation analysis of behaviors, relative abundance of gut microbiota, pro‐inflammatory cytokines levels, and gene expression of tight junction proteins in hippocampus in female mice. (A) Levels of IL‐1β and IL‐6, and gene expressions of cldn‐8, ocln, and zo‐1 in hippocampus were significantly correlated with anxiety‐like behavior. (B) Relative abundance of gut microbiota in phylum and genus levels were significantly correlated with anxiety‐like and depression‐like behaviors, IL‐1β levels, and zo‐1, ocln, cldn‐5, and cldn‐8 gene expressions in hippocampus. *p < 0.05, **p < 0.01, ***p < 0.001; Spearman's rank correlation.

We selected pro‐inflammatory cytokines and mRNA of tight junction proteins that were different between three groups in female mice and explored whether the levels of these molecules significantly co‐varied with behavioral outcomes by Spearman's rank correlation. We found that IL‐1β, IL‐6, and zo‐1 mRNA levels in the hippocampus positively correlated with anxiety‐like behavior, while ocln and cldn‐8 mRNA levels negatively correlated with anxiety‐like behavior (Figure 7A).

In female mice, the abundance of two phyla and six genera changed significantly after 3‐week experiment in the CRS group, but not in the CON or CP groups. We found that the abundance of *Alloprevotella genus* and *Parabacteroides genus* positively correlated with distance traveled in center zone in OFT, while *Bifidobacterium genus* and *Ruminococcaceae_UCG‐014 genus* negatively correlated with immobile time in FST. *Alloprevotella genus*, *Parabacteroides genus*, and *Bacteroidetes phylum* negatively correlated with IL‐1β level in the hippocampus, but *Lachnospiraceae_NK4A136_group genus*, *Roseburia genus*, and *Firmicutes phylum* showed the opposite effects. In addition, *Alloprevotella genus* and *Parabacteroides genus* positively correlated with ocln and cldn‐8 mRNA levels, and negatively correlated with zo‐1 mRNA levels in the hippocampus (Figure 7B).

## 4 DISCUSSION

Clinical studies have showed that probiotics had antidepressive and anxiolytic effects, but the treatment outcome of prebiotics on depression and anxiety was inconsistent.^36^ Previous studies indicated that prebiotics showed anxiolytic effects in patients with irritable bowel syndrome (IBS),^37^, ^38^ but not in healthy individuals,^39^ and no antidepressive effects in patients with depression.^40^ We speculated that antidepressive and anxiolytic effects of prebiotics might be related with gastrointestinal symptoms. Many animal studies also indicated that prebiotics, such as BGOS, FOS, and yacon oligosaccharides (YOs), could reduce depression‐like and anxiety‐like behaviors.^11^, ^41^, ^42^ However, we only observed that the anxiolytic effects of prebiotics on CRS‐induced mood symptoms in both male and female mice. This inconsistence in findings might be attributed to the difference in prebiotics intervention formula and animal models for depression, because different animal models for depression showed various depressive phenotypes and transcriptomic profiles in brains.^43^ In addition, comparing with male mice, we found that anxiety in female mice was more easily alleviated by prebiotics. Of course, the result was only found in CRS‐induced depression model. Future studies using various animal models for depression are warranted to confirm the effect of prebiotics on depression in a sex‐specific manner.

At baseline, we found that there was a clear separation in gut microbial diversity between female and male mice, similar to a previous study.^44^ Alpha diversity in female was higher than that in male, but chronic stress could induce lower alpha diversity in male, which were not reversed by prebiotics.

Interestingly, the difference also existed in changes of relative abundance of gut microbiota induced by CRS between female and male, and prebiotics partially mitigated microbiota changes. At the phylum level, *Firmicutes* and *Bacteroidetes* were the dominant microbiota in both female and male mice. Change in *Firmicutes/Bacteroidetes* ratio was considered as a maker of gut dysbiosis.^45^ We found that 3‐week CRS treatment increased the ratio of *Firmicutes/Bacteroidetes* in female mice, that could be prevented by prebiotics administration, but no change was observed in male mice. At the genus level, the increase in abundance of *Lachnospiraceae_NK4A136_group* (the dominant flora genus) induced by chronic stress was mitigated by prebiotics administration in female, but not in male. Although *Lachnospiraceae* could produce short‐chain fatty acids that were commonly considered to be beneficial for human health, many studies showed that increased abundance of *Lachnospiraceae* was associated with many metabolic diseases and depression.^46^ *Parabacteroides* was a gram‐negative anaerobic bacteria, which modulated and improved inflammation, intestinal barrier function, metabolize, and circadian rhythm.^47^, ^48^, ^49^, ^50^ In our study, we found that prebiotics prevented the reduced abundance of *Parabacteroides* caused by CRS in female, but not in male. By correlation analysis, we found that *Parabacteroides* negatively correlated with anxiety‐like behavior. These results were also observed in *Alloprevotella*. We speculated that prebiotics administration prevented anxiety‐like behavior in female by *Parabacteroides* and *Alloprevotella*. FOS and GOS were traditionally associated with the increase in beneficial bacteria including *Bifidobacterium* and *Lactobacillus*.^34^ In previous human and animal studies, lower abundance of two bacteria was found in depression patients compared with healthy individuals,^51^ and *Bifidobacterium* or *Lactobacillus* administration alleviated depression‐like behavior in rodents.^9^, ^52^ Contrary to our expectation, however, 3‐week chronic stress did not decrease the abundance of *Lactobacillus* in female and there was no difference among three groups after experiment in male mice. The results in *Bifidobacterium* were similar to previous studies, showing chronic stress could decrease the abundance of *Bifidobacterium* in both female and male mice, and prebiotics administration prevented the change. In the results of correlation analysis, *Bifidobacterium* negatively correlated with immobile time in FST, which indicated that *Bifidobacterium* might alleviated depression‐like behavior, in consistency with previous studies.^9^, ^52^ The results in gut microbiota showed that female mice were more vulnerable to stress than male, and prebiotics could prevent some flora changes induced by chronic stress in a sex‐specific manner. Several previous studies showed that sex hormones might interact with gut microbiota.^53^ We suspected that the different changes between female and male in gut microbiota caused by stress and prebiotics might be related with sex hormone, but further studies were needed to test.

Both gut microbiota and stress affected gut barrier and BBB function,^17^, ^54^, ^55^ dysfunction of these barriers contributed to the pathogenesis of depression.^56^, ^57^ In our study, 3‐week CRS treatment mainly increased gene expression of tight junction proteins and muc‐2, and decreased numbers of goblet cells in colon of female mice, but not in male. Higher ocln and zo‐1 mRNA levels in intestine were also observed in female rats after chronic isolation and male rats after subacute stress.^16^, ^29^ Increased gene expressions of tight junction proteins might be a compensatory upregulation after gut barrier dysfunction. Previous studies found that muc‐2 was the key molecule for the formation of mucus barriers.^58^ Mucus barrier built by muc‐2 could separate microorganisms from intestinal mucosal epithelium and avoid intestinal inflammatory reaction.^59^ As we expected, pro‐inflammatory cytokines levels in the colon were increased after chronic stress only in female, which was similar to gut barrier function, indicating that gut barrier function and inflammatory response were more vulnerable to chronic stress in female. It was well known that gut microbiota was closely related to gut mucosal barrier and inflammatory response.^60^ The susceptibility of dysfunction in gut mucosal barrier and inflammatory response to chronic stress in female might be related to the changes of gut microbiota to chronic stress. In addition, these changes caused by 3‐week CRS in female were alleviated by FOS + GOS administration, further suggesting that gut microbiota played an important role in barrier function and inflammation in colon. And as regulators of gut microbiota, FOS + GOS could effectively prevented gut barrier dysfunction and inflammation caused by stress in female.

One previous study found that 3‐week CRS did not alter BBB permeability in hippocampus of male mice,^61^ but other studies showed opposite results.^55^, ^62^, ^63^ In our study, 3‐week CRS treatment downregulated ocln, cldn‐2, and cldn‐8 mRNA levels, and upregulated zo‐1 mRNA levels in hippocampus of female, but not in male. To further evaluate BBB function, we conducted an in vivo assay to test BBB permeability based on intravenous injection of Evans Blue in mice and found stress‐induced BBB dysfunction in hippocampus could be improved by prebiotics in both female and male mice. Many studies showed that inflammatory factors, such as IL‐1β, IL‐6, and TNF‐α, could disrupted BBB integrity.^64^, ^65^ Our study also found 3‐week CRS increased IL‐1β and IL‐6 levels in hippocampus of female and male, and the changes were alleviated by prebiotics, further indicating the correlation between neuro‐inflammation and BBB function. The results of inflammation and BBB function in hippocampus showed that stress and prebiotics had the same effects on female and male mice. However, the improvement of prebiotics on inflammation and BBB dysfunction in hippocampus caused by stress led to a reduction in anxiety‐like behavior in female, but not in male mice, indicating female mice were more vulnerable to the changes of BBB function and inflammation levels in hippocampus.

Reports on increased BBB permeability and decreased expression of tight junction proteins in germ‐free and antibiotic‐treated mice showed that gut microbiota had regulatory effects on BBB function.^23^, ^66^ Interestingly, prebiotics administration during 3‐week CRS could alleviated inflammation and gut and brain–blood barriers dysfunction caused by CRS, and these changes correlated with anxiety‐like behavior in female. We also found that *Parabacteroides* and *Alloprevotella* negatively correlated with pro‐inflammatory cytokines levels and anxiety‐like behavior, and positively correlated with gene expressions of tight junction proteins in hippocampus. As aforementioned, increasing abundance of *Parabacteroides* by prebiotics administration could modulate inflammation and increase expression of tight junction proteins in colon.^47^ These results showed that *Parabacteroides* might prove anxiety‐like behavior by alleviating colon‐ and neuro‐inflammation, and improving gut and brain–blood barrier function.

## 5 CONCLUSION

In conclusion, this study revealed that prebiotics could alleviate anxiety‐like behavior caused by stress in a sex‐specific manner. Gut microbiota dysbiosis, intestinal barrier, and BBB dysfunction induced by stress were more vulnerable in female when compared to male. Prebiotics may alleviate anxiety in female via changing the gut microbiota composition, maintaining intestinal homeostasis, improving intestinal battier and BBB function, and mitigating peripheral and neuro‐inflammation.

## CONFLICT OF INTEREST

The authors declared no competing financial interest.

## Supporting information


Appendix S1

## Data Availability

The data that support the findings of this study are available from the corresponding author upon reasonable request.
