# Supplementary material for: Sex difference in prebiotics on gut and blood–brain barrier dysfunction underlying stress‐induced anxiety and depression
Source: CNS Neurosci Ther. 2023 Jan 17;29(Suppl 1):115–28. doi: 10.1111/cns.14091 (PMC10314104; doi:10.1111/cns.14091)
Supplement: Supplementary file 1 — Appendix S1 [file CNS-29-115-s001.docx]

**Supplementary Table 1**. Primers for β-actin, zo-1, occluding (ocln), claudin-1(cldn1), claudin-2, claudin-5, claudin-8 and muc-2.

Genes Forward primer (5’-3’) Reverse primer (5’-3’)

zo-1 CTGGTGAAGTCTCGGAAAAATG CATCTCTTGCTGCCAAACTATC

ocln TGCTTCATCGCTTCCTTAGTAA GGGTTCACTCCCATTATGTACA

cldn-1 AGATACAGTGCAAAGTCTTCGA CAGGATGCCAATTACCATCAAG

cldn-2 GGTTCCTGACAGCATGAAATTT GCCATCATAGTAGTTGGTACGA

cldn-5 GTGGCACTCTTTGTTACCTTG GATCATAGAACTCGCGGACAA

cldn-8 TGTCTGCCTTCATCGAAAGTAA GGCATGCCTCATACAATTCATC

muc-2 TGCTGACGAGTGGTTGGTGAATG TGATGAGGTGGCAGACAGGAGAC

β-actin CTACCTCATGAAGATCCTGACC CACAGCTTCTCTTTGATGTCAC


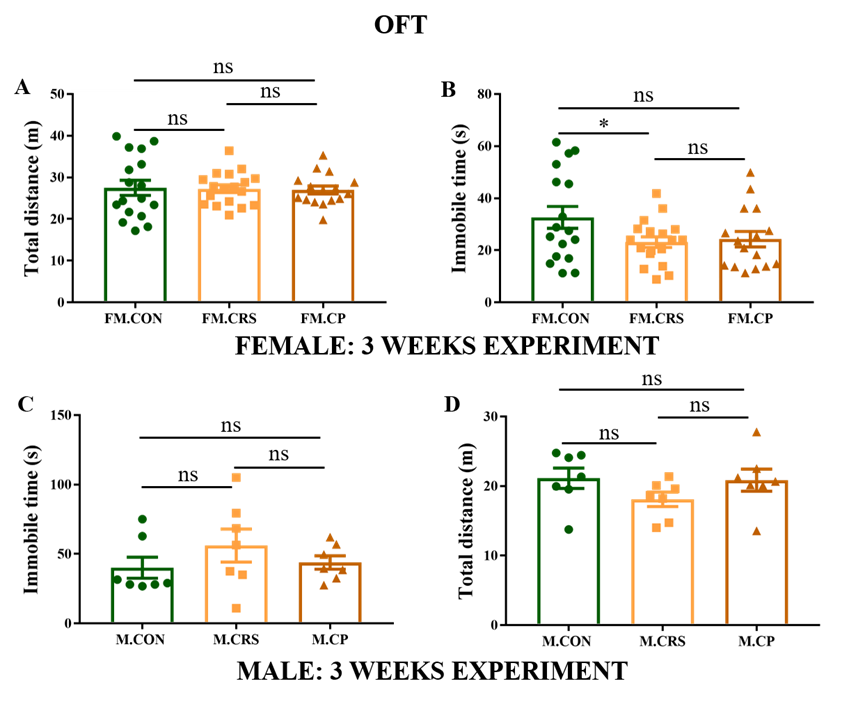

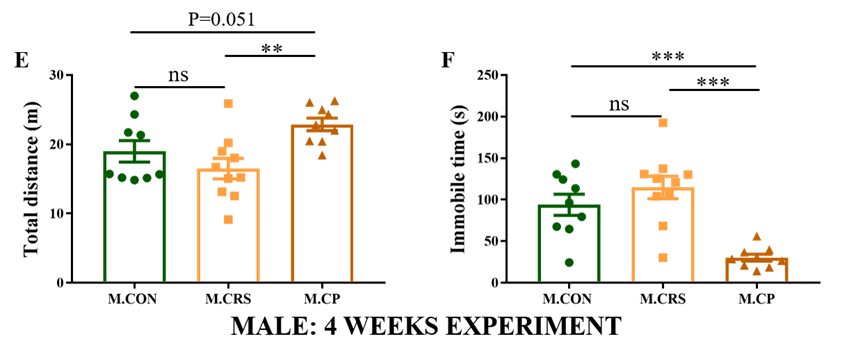


**Supplementary Figure 1.** Behavioral phenotypes of OFT in 3 and 4-week experiment.

(A, B) Total distance and immobile time of OFT in female mice in 3-week experiment. (C, D) Total distance and immobile time of OFT in male mice in 3-week experiment. (E, F) Total distance and immobile time of OFT in male mice in 4-week experiment.

* p<0.05, ** p<0.01, *** p<0.001; Independent Sample T-test analysis; data represent mean±SEM.


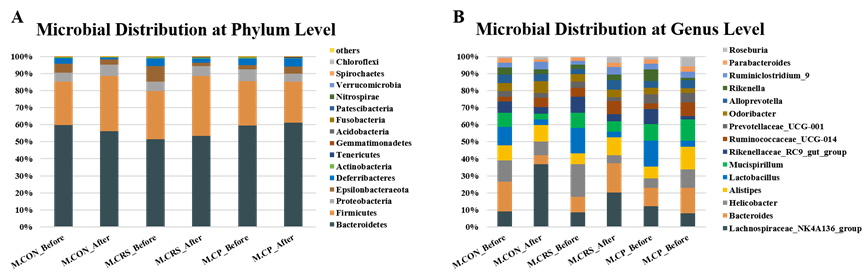

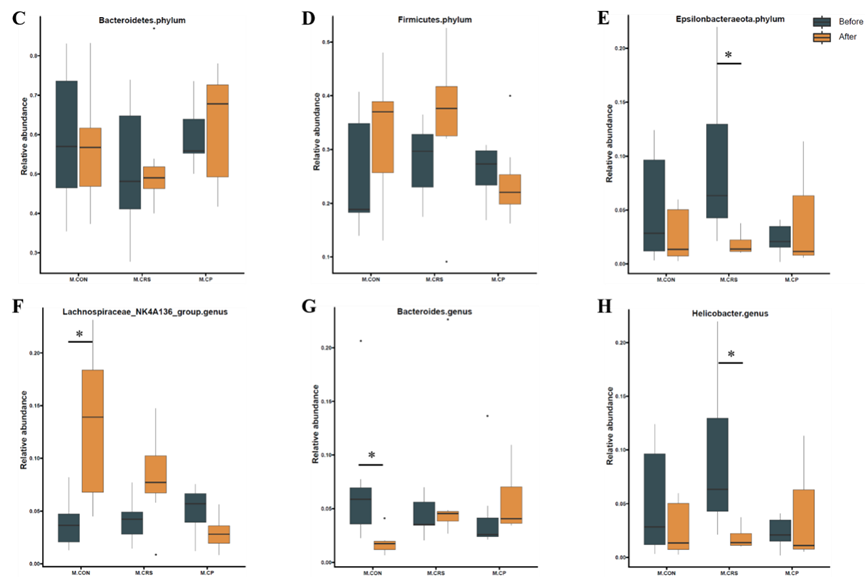

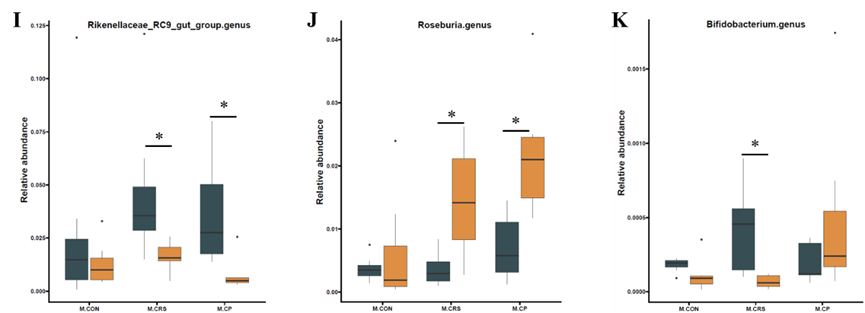


**Supplementary Figure 2.** Microbial distribution and relative abundance of selected phylum and genus among male mice groups.

(A, C-E) Microbial distribution at phylum level. *Bacteroidetes* and *Firmicutes* were the main components of microbiota, but relative abundance of these two genera did not change significantly in all groups after 3-week experiment. Decreased abundance of *Epsilonbacteraeota* was observed after 3-week CRS treatment, but not in the CON or CP groups. (B, F, G) Microbial distribution at genus level. *Lachnospiraceae_NK4A136_group* and *Bacteroides* were the predominant gut bacteria, and relative abundance of these two genera changed significantly in the CON group, but not in the CRS or CP group after 3-week experiment. (H-K) At genus level, 3-week CRS treatment induced a decrease in the abundance of *Helicobacter*, *Rikenellaceae_RC9_gut_group* and *Bifidobacterium*, and increased the abundance of *Roseburia*. Among these genera, there were no differences in the CON group. Similar changes in *Rikenellaceae_RC9_gut_group* and *Roseburia* were observed in the CP group.

* p<0.05; Kruskal–Wallis test and Mann-Whitney test; n=7-8.
